# Impaired repair properties of endothelial colony‐forming cells in patients with granulomatosis with polyangiitis

**DOI:** 10.1111/jcmm.17531

**Published:** 2022-09-02

**Authors:** Ana Paula Toledo Del Rio, Jéssica O Frade‐Guanaes, Stephanie Ospina‐Prieto, Bruno K L Duarte, Manoel Barros Bertolo, Margareth C Ozelo, Zoraida Sachetto

**Affiliations:** ^1^ Rheumatology Discipline, School of Medical Sciences University of Campinas (UNICAMP) Campinas Brazil; ^2^ Hemocentro UNICAMP, School of Medical Sciences University of Campinas (UNICAMP) Campinas Brazil

**Keywords:** anti‐neutrophil cytoplasmic antibody‐associated vasculitis, endothelial colony‐forming cells, endothelial progenitor cells, granulomatosis with polyangiitis

## Abstract

In patients with ANCA‐associated vasculitis, interactions between neutrophils and endothelial cells cause endothelial damage and imbalance. Endothelial colony‐forming cells (ECFCs) represent a cellular population of the endothelial lineage with proliferative capacity and vasoreparative properties. This study aimed to evaluate the angiogenic capacity of ECFCs of patients with granulomatosis with polyangiitis (GPA). The ECFCs of 13 patients with PR3‐positive GPA and 14 healthy controls were isolated and characterized using fluorescence‐activated cell sorting, capillary tube formation measurement, scratching assays and migration assays with and without plasma stimulation. Furthermore, three patients with active disease underwent post‐treatment recollection of ECFCs for longitudinal evaluation. The ECFCs from the patients and controls showed similar capillary structure formation. However, the ECFCs from the patients with inactive GPA exhibited early losses of angiogenic capacity. Impairments in the migration capacities of the ECFCs were also observed in patients with GPA and controls (12th h, *p* = 0.05). Incubation of ECFCs from patients with GPA in remission with plasma from healthy controls significantly decreased migration capacity (*p* = 0.0001). Longitudinal analysis revealed that treatment significantly lowered ECFC migration rates. This study revealed that ECFCs from the patients with PR3‐positive GPA in remission demonstrated early losses of tube formation and reduced migration capacity compared to those of the healthy controls, suggesting impairment of endothelial function.

## 1 INTRODUCTION

Granulomatosis with polyangiitis (GPA) is a recurrent severe antineutrophil cytoplasmic antibody (ANCA)‐associated vasculitis (AAV) characterized by the presence of pauci‐immune necrotizing vasculitis and autoantibodies.^1^ The pathogenesis of ANCA‐associated vasculitis is multifactorial, involving numerous immune cells. ANCAs may play central roles in the pathogenesis of anti‐proteinase 3 (PR3)‐positive GPA, and neutrophil activation is required for loss of endothelial integrity.^2^, ^3^, ^4^, ^5^ Despite treatment, disease recurrence is very common, with increased cardiovascular risk and high morbidity and mortality rates.^6^, ^7^

Endothelial progenitor cells (EPCs), identified by Asahara et al. in 1997, have a proven role in endothelial repair under physiological and pathological conditions, with significant involvement in postnatal vasculogenesis.^8^, ^9^ Currently, there is a considerable controversy regarding the definition of EPCs because this term includes populations of myeloid angiogenic cells (MACs) that are not members of the endothelial lineage. Different approaches have been described for the differentiation of EPCs based on flow cytometry of peripheral blood samples and peripheral blood monocyte isolation and culture.^10^, ^11^ The number of EPCs, as measured by flow cytometry, has been described as potential evidence of endothelial damage in ischemic diseases, and their proliferative and regenerative functions have been demonstrated in animal studies and human clinical trials.^12^

Endothelial colony‐forming cells (ECFCs) represent a cellular population of the endothelial lineage, with stable culture‐wild phenotypes in vitro, high proliferative capacity and vasoreparative properties.^9^, ^13^, ^14^ Impaired vascular repair due to alterations in the angiogenic function of ECFC has also been observed in patients with vascular diseases.^15^ Their capacity for self‐renewal and angiogenesis makes ECFCs a subject of great interest in cell‐based regenerative therapy studies.^16^

Although endothelial damage can significantly impact AAV, few studies have explored the effects of an impaired vascular repair system. Previous studies suggest that endothelial repair dysfunction in patients with AAV are caused by changes in circulating EPCs.^17^, ^18^, ^19^ Previous studies examining the cultures of endothelial cells from patients with AAV showed that they have lower proliferative capacities than those from healthy patients.^19^, ^20^, ^21^ However, it should be noted that only one of these studies, specifically that of Wilde et al.^21^ performed experiments involving ECFCs.

To date, no studies have explored the angiogenic capacity of ECFCs in patients with AAV. Identifying the characteristics and functions of such cells is essential to better understand the role of the endothelium in the etiopathogenesis of AAV and to develop potential therapeutic targets. In this study, ECFCs isolated from peripheral blood and cultured in vitro were used as study models. This study aimed to evaluate migration and angiogenesis of ECFCs in patients with GPA.

## 2 METHODS

### 2.1 Patients and controls

Patients diagnosed with GPA were enrolled in the study. All patients were recruited from the Rheumatology Unit of the State University of Campinas, Brazil. The controls included healthy volunteers and human umbilical vein primary endothelial cells (HUVECs). This study was conducted in accordance with the Declaration of Helsinki and approved by the local ethics committee of the Faculty of Medical Sciences of the State University of Campinas – UNICAMP (CAAE number:38488914.4.0000.5404). All participants provided written informed consent before the study.

The inclusion criteria were as follows: age >18 years at the onset of symptoms, newly diagnosed but not yet treated active patients or GPA patients in remission who were undergoing follow‐up without immunosuppression for at least 24 months, intake of prednisone ≤10 mg/day, normal hematimetric indices and no infections for at least 6 months.

Granulomatosis with polyangiitis was diagnosed according to the ACR criteria (1990) and the International Consensus Conference on the nomenclature of systemic vasculitis (Chappel Hill 2013). Disease activity was measured according to the Birmingham Vasculitis Activity Score (BVAS).^22^, ^23^ Active disease was defined as the recent onset of clinical manifestations of AAV‐related disease requiring immunosuppressive therapy. Remission was defined as the absence of clinical disease activity and zero BVAS score for at least 24 months.^20^, ^24^

### 2.2 Isolation of ECFCs

After peripheral blood collection, isolation and expansion, ECFCs were prepared on in vitro plates for angiogenesis and migration assays. ECFCs were cultured according to the protocol described by Lin et al., with some modifications.^25^, ^26^ Briefly, 45 ml of peripheral blood from each participant was drawn into tubes containing sodium heparin anticoagulant (BD Vacutainer). To isolate buffy coat mononuclear cells, the samples were subjected to density gradient centrifugation for 30 min with Ficoll‐Paque reagent TM Plus 1077 (GE Healthcare) after dilution (1:1) in phosphate‐buffered saline (PBS). The cell pellets were washed with PBS and resuspended in endothelial basal medium (EBM‐2) (Lonza, Walkersville). Approximately 1 × 10^7^ cells were cultured in 12‐well plates previously treated with rat tail collagen type I (50 μg/L) (Sigma‐Aldrich) in 1.5 ml EBM‐2 supplemented with endothelial growth media (EGM)‐2 SingleQuots kit (Lonza), which contained 10% foetal bovine serum (FBS). The plates were incubated at 37°C under 5% CO_2_, and the culture media were refreshed daily for 7 days and every other day thereafter. Upon reaching confluence, adherent cells were removed via trypsinization (Gibco Invitrogen) until the third passage after approximately 40 days of culture. After the third passage, the ECFCs were characterized using fluorescence‐activated cell sorting (FACS) and sent for cryopreservation.

### 2.3 FACS analysis

The characterization of ECFCs was performed using specific antibodies conjugated to fluorochromes (fluorescein isothiocyanate [FITC], phycoerythrin [PE] and peridin chlorophyll protein [PerCP]), which detect the following specific endothelial surface markers: anti‐CD31‐FITC, clone MBC 78.2 (Invitrogen); anti‐CD144‐PE, clone TEA 1/31 (Beckman Coulter); anti‐CD146PE, clone 128,018 (R&D Systems); anti‐VEGF R2/KDR‐PE, clone 89,106 (R&D Systems); anti‐CD34‐FITC, My10 clone (BD); anti‐CD45‐PerCP, clone 2D1 (BD); anti‐CD133‐APC, clone AC133 (Miltenyi Biotech). The tubes were incubated for 30 min at 4°C, protected from light, washed with PBS, centrifuged at 450 *g* for 5 min (RT) and resuspended in 300 μl of PBS for the acquisition of 10,000 events on a flow cytometer (FACS Calibur, Immunofluorometry Systems). Data analysis was performed using the BD FACS DIVA software (v.7.0; San Jose, CA, USA). The cells were considered ECFCs if they tested positive for CD31, CD144, CD146 and KDR markers, negative for CD45 and CD133, and exhibited decreased CD34 expression.

### 2.4 Matrigel assay

To assess endothelial tube formation in vitro, ECFCs and HUVECs were cultured in a 24‐well plate (10 × 10^4^ cells/well) in a basement‐membrane‐like substrate (Matrigel™, BD Biosciences) for 24 h.^27^ The cells were photographed after 15 and 24 h of incubation using an inverted microscope (Olympus IX81, Olympus) coupled with a digital camera (Olympus DP72, Olympus) at 4× magnification in the phase‐contrast mode. Characterization of capillary‐like structures, such as extremities, junctions, nodes, meshes and segments, was performed using ImageJ software (National Institutes of Health, Bethesda). All experiments were performed in triplicate for each patient, control and HUVEC sample.

### 2.5 Migration assay

To assess cell migration, ECFCs were grown in 24‐well plates coated with complete EBM‐2 medium supplemented with collagen until confluent monolayers were formed. Scratch assays were performed by introducing a thin ‘wound’ by scratching the bottom of the well with a sterile 200 μl pipette tip.^28^ The plates with fresh media were photographed at 0 min and every 60 min thereafter for 24 h using an inverted fluorescence microscope (Zeiss LSM780‐NLO, Carl‐Zeiss) at 10× magnification, collecting three images per well at each time point. The obtained images were analysed using ImageJ software. The distance between cells in multiple scratched areas in each culture well was measured as the rate of wound closure. All experiments were performed in triplicate for each patient, control and HUVEC sample. ECFCs from three patients with active disease were re‐collected after 6 months of treatment with cyclophosphamide for longitudinal evaluation.

### 2.6 Plasma influence on migration in vitro

To assess the influence of plasma on cell migration, we reproduced an environment conducive to cell migration by incubating ECFCs with plasma overnight. For this procedure, EBM‐2 medium without FBS was supplemented with 10% plasma from a patient with active GPA or a healthy control. Migration assays were performed under these conditions.

### 2.7 Statistical analysis

Statistical analyses were performed using SAS (Statistical Analysis System) for Windows (v.9.4. SAS Institute Inc., 2002–2012). Variables are expressed as means with standard deviations (SDs). anova was used for repeated measurements over time in the Matrigel assay and to compare three or more groups in the plasma migration assays. The Kruskal–Wallis test was performed to compare the groups in the migration assay, with Dunn's test used for multiple comparisons. Statistical significance was set at *p* ≤ 0.05. Graphics were created using GraphPad Prism, v.6.00 for Windows (GraphPad Software Inc.).

## 3 RESULTS

### 3.1 Isolation and culture of human ECFCs

Peripheral blood samples were collected from 13 patients with GPA and 14 healthy controls. All patients with GPA tested positive for anti‐PR3 antibodies at the time of diagnosis. Cell cultures were successful in eight (62%) patients with GPA (five with disease activity and three in remission) and eight (57%) controls, with median ages of 29 and 39 years, respectively.

**FIGURE 1:**
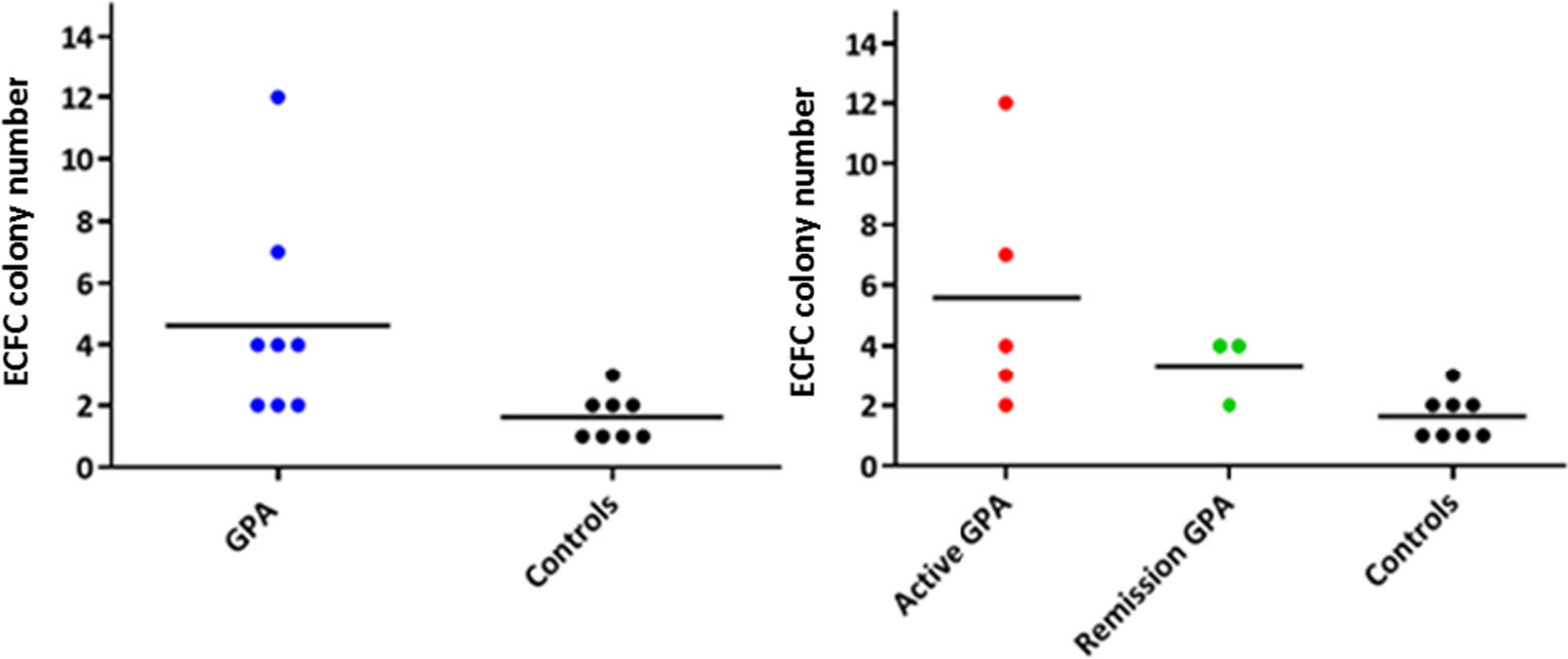
Dotplot showing successful ECFC colony number isolation of GPA active and remission patients and controls.

On an average, the first ECFC colonies from patients with GPA and controls appeared on the 15th day. There were no significant differences in the number of colonies among the control, active disease and remission groups at the end of the third passage. However, one patient with active GPA presented a colony count that was four times higher than that of the others. Patient data and ECFC culture results are summarized in Table 1 and Figure 1.

**TABLE 1:** Patient data and ECFC culture characteristics

	Controls		GPA	
	All	ECFC isolation	All	ECFC isolation
Patients, *n* (%)	14 (100)	8 (57)	13 (100)	8 (62)
Median age, years (min‐max)	30 (22–53)	29 (22–53)	43 (21–72)	39 (21–72)
Female/Male, *n* (%)	8(57) /6(43)	4(50) /4(50)	8(62)/ 5(38)	4(50)/ 4(50)
Leukocytes 106/ml, mean (min‐max)	14.5 (6–27.9)	13.5 (7.6–27.9)	8.2 (3.1–23.2)	10.2 (4.6–23.2)
ECFC emergency, mean day (min‐max)	‐	15 (11–22)	‐	15 (9–22)
Colony, mean (min‐max)	‐	1.6 (1–3)	‐	4.8 (1–12)

For longitudinal evaluation, samples were collected for the isolation and culture of ECFCs at diagnosis and after inducing remission with cyclophosphamide in three patients. Pre‐treatment isolation was successful in all three cases, and in only one case after immunosuppression. Successful immunosuppression resulted in the highest number of pre‐treatment colonies.

### 3.2 Characterization of GPA and control ECFCs

**FIGURE 2:**
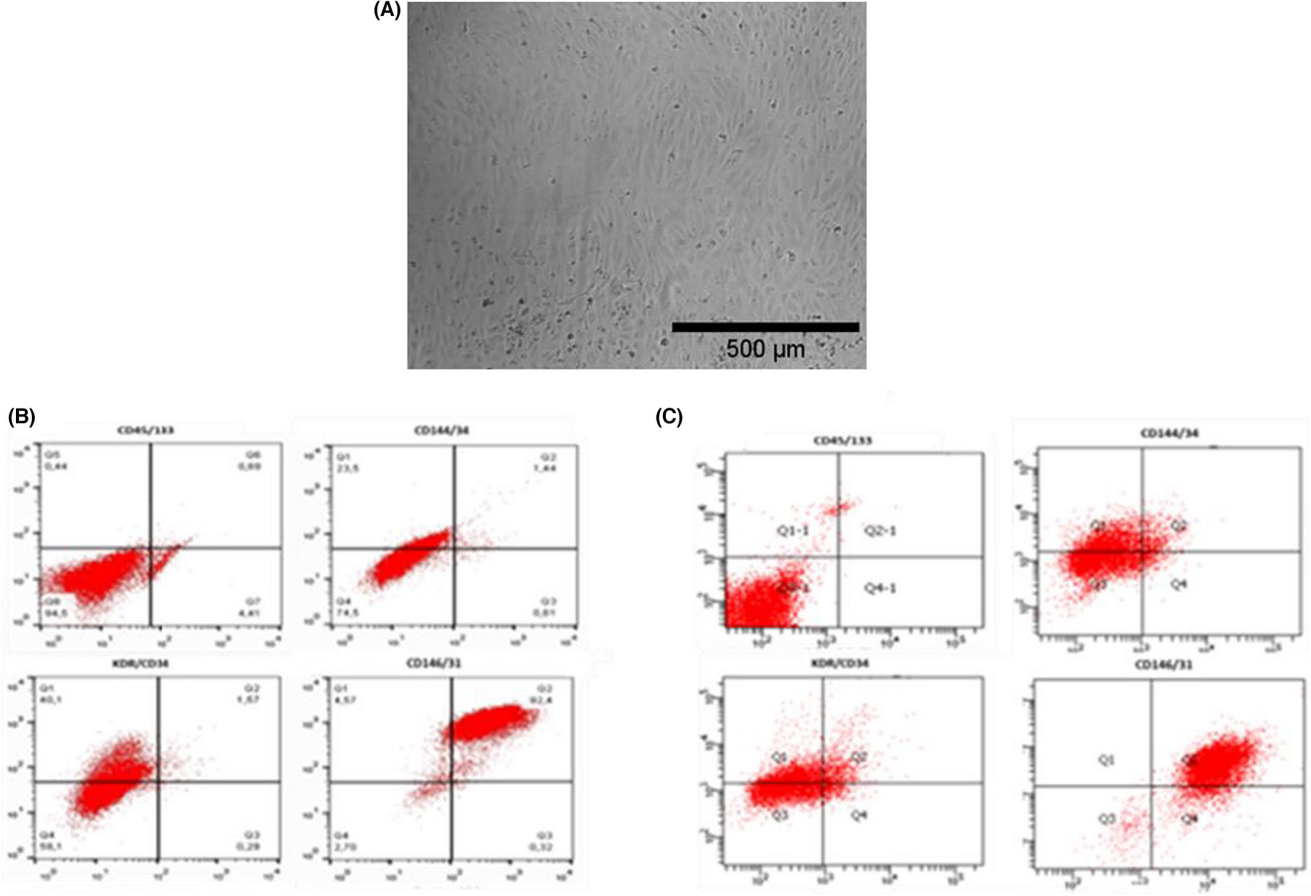
(A) Characteristic cobblestone morphology of confluent ECFCs on a collagen plate from a patient with GPA. (B) FACS analysis: the histograms demonstrate that the ECFCs were positive for the following endothelial cell markers: CD31 (PECAM), KDR (VEGFR), CD144 and CD146; and negative for the following monocyte markers: CD133 and CD45; and showed decreased CD34 levels. Histograms are based on culture of the ECFCs from a patient with GPA. (C) Histograms based on culture of the ECFCs from a healthy control. This Figure is representative of patients with GPA and controls.

The ECFCs of patients with GPA exhibited a typical endothelial cobblestone morphology. FACS analysis confirmed that the ECFCs were endothelial cells expressing the following specific markers: CD31 (PECAM), KDR (VEGFR), CD144 (VE‐Kadhrin) and CD146. As expected, ECFCs were negative for CD133 and CD45 (monocyte markers) and showed decreased expression of CD34. Control ECFCs demonstrated similar results (Figure 2).

### 3.3 Efficiency of ECFCs in forming capillary‐like structures

**FIGURE 3:**
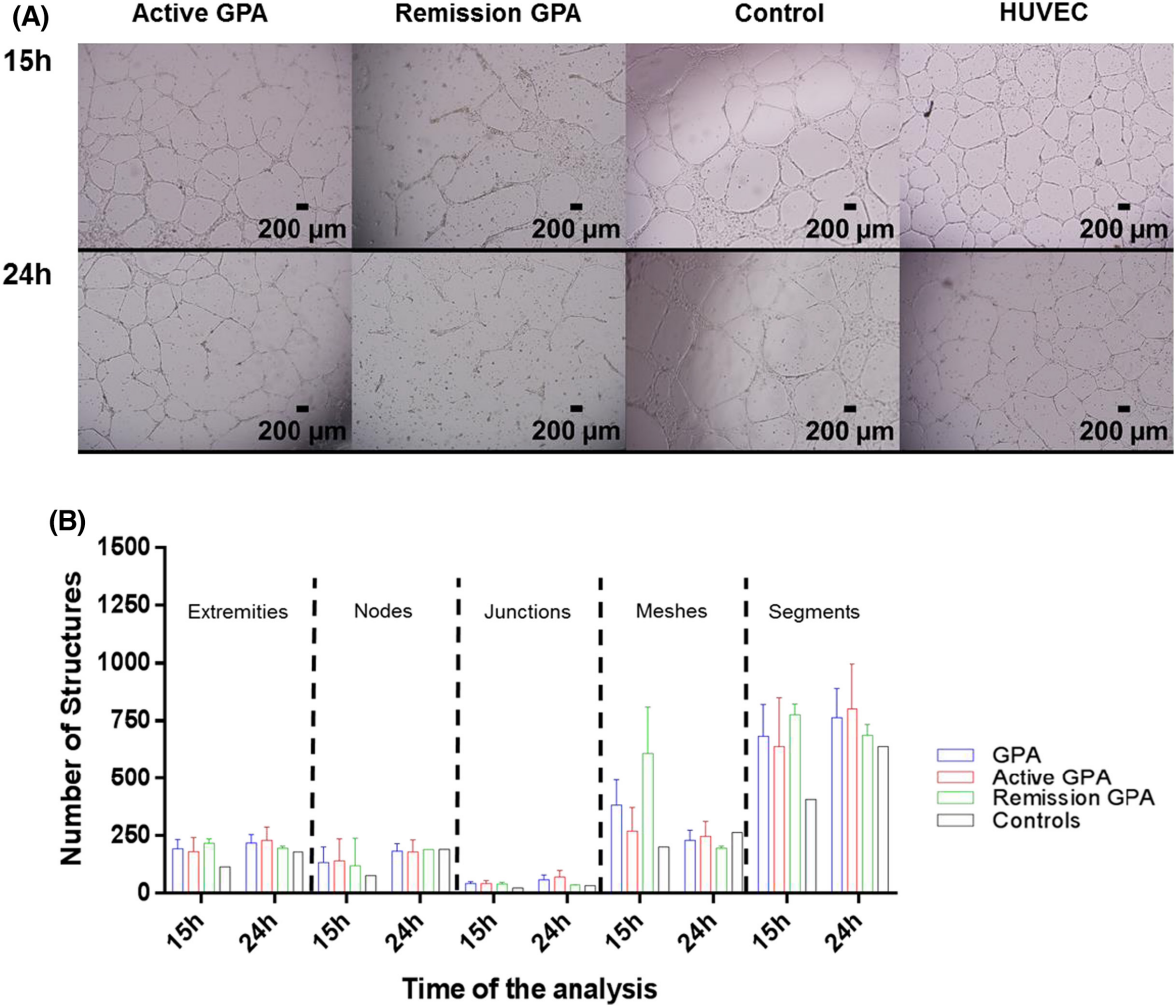
Matrigel assay: (A) ECFCs from patients with active GPA and patients in remission formed capillary‐like structures (J = junctions, S = segments, M = meshes, E = extremities and N = nodes) comparable with those observed in ECFCs from healthy controls. ECFCs from patients with GPA, controls and HUVECs were able to form capillary‐like structures; (B) Angiogenesis analysis showed a greater angiogenic capacity in ECFCs from patients with GPA, but this was not statistically significant. A progressive increase in such structures formed after 24 h was observed in patients with active GPA, controls and HUVECs, but not in patients in remission. The data are representative of patients with GPA and controls.

Matrigel assays revealed similar numbers of structures (extremities, junctions, nodes, meshes and segments) formed by ECFCs from patients with GPA compared with the control group (*p* = 0.18, *p* = 0.57, *p* = 0.49, *p* = 0.76 and *p* = 0.82, respectively). When classified according to the presence or absence of disease activity, it was observed that only patients in remission exhibited progressive decreases in the numbers of most structures after 15 h (Figure 3).

### 3.4 Migration assay

**FIGURE 4:**
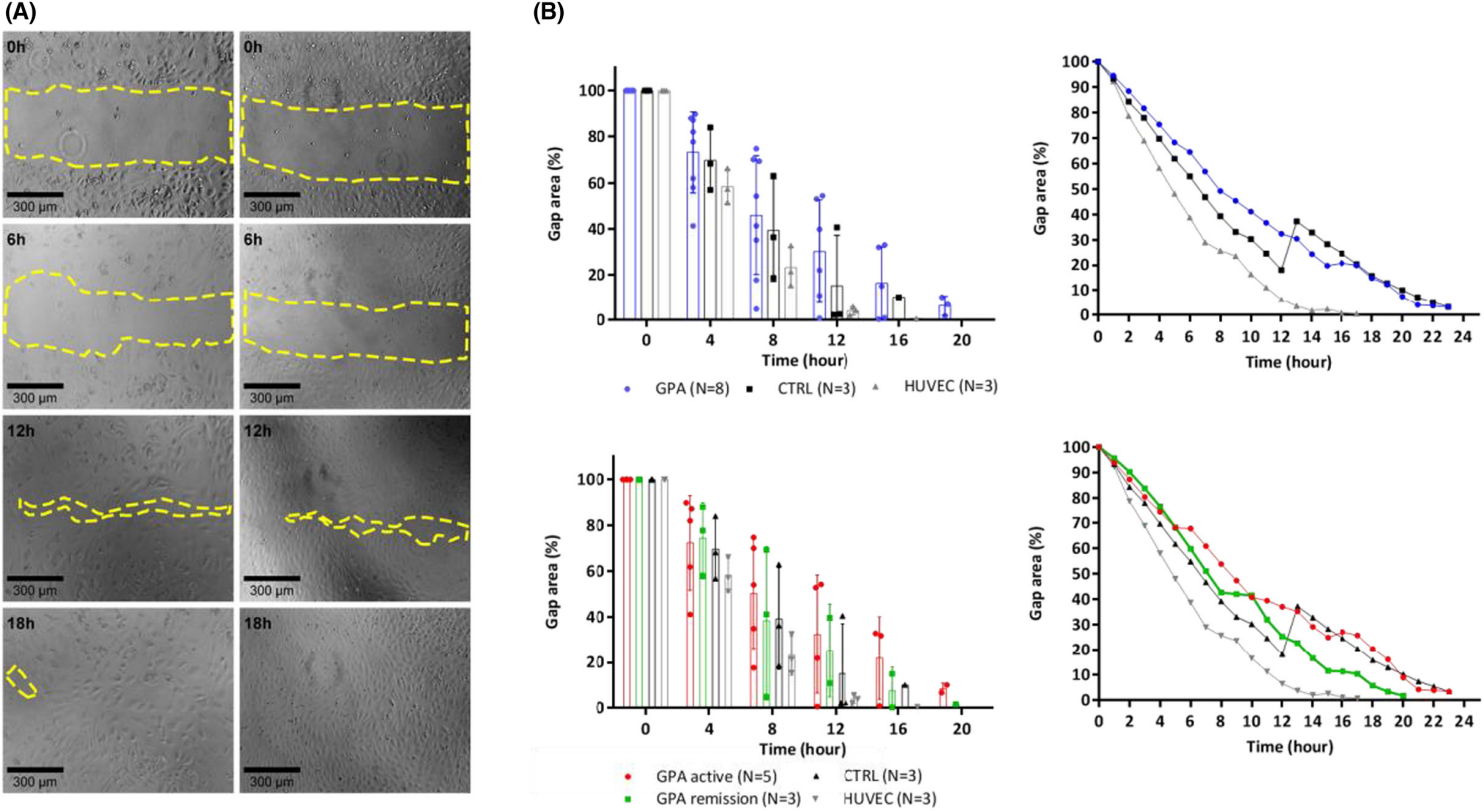
Measurement of cell migration by in vitro scratch assay: (A) ECFC migration photographed hourly for 24 h after scratching. The rate of migration was measured by quantifying the total area that the ECFC moved from the edge towards the centre of the scratched (yellow dotted line). The photographs and data are representative of patients with GPA and controls. (B) There were no significant differences in migration capacities after overnight incubation with EBM‐2 (37°C), between ECFCs of patients with GPA and controls. There was a similar confluence behaviour considering individual samples and means of the groups.

Migration assays were performed using the scratching method, in which the values from each group were collected and compared every 4 h for a total of 24 h until the gap area was closed. There were no significant differences in the migration capacities between the ECFCs of patients with GPA and controls (12th h, *p* = 0.05). This was also observed even when patients were divided according to the presence or absence of disease activity (12th h, *p* = 0.08) (Figure 4).

Additionally, no differences were found between the averages of the lowest confluence percentages that each group (GPA, controls and HUVECs) reached by 24 h and the mean time that each group took to close the gap area (*p* = 0.20, *p* = 0.91).

### 3.5 Longitudinal analysis

**FIGURE 5:**
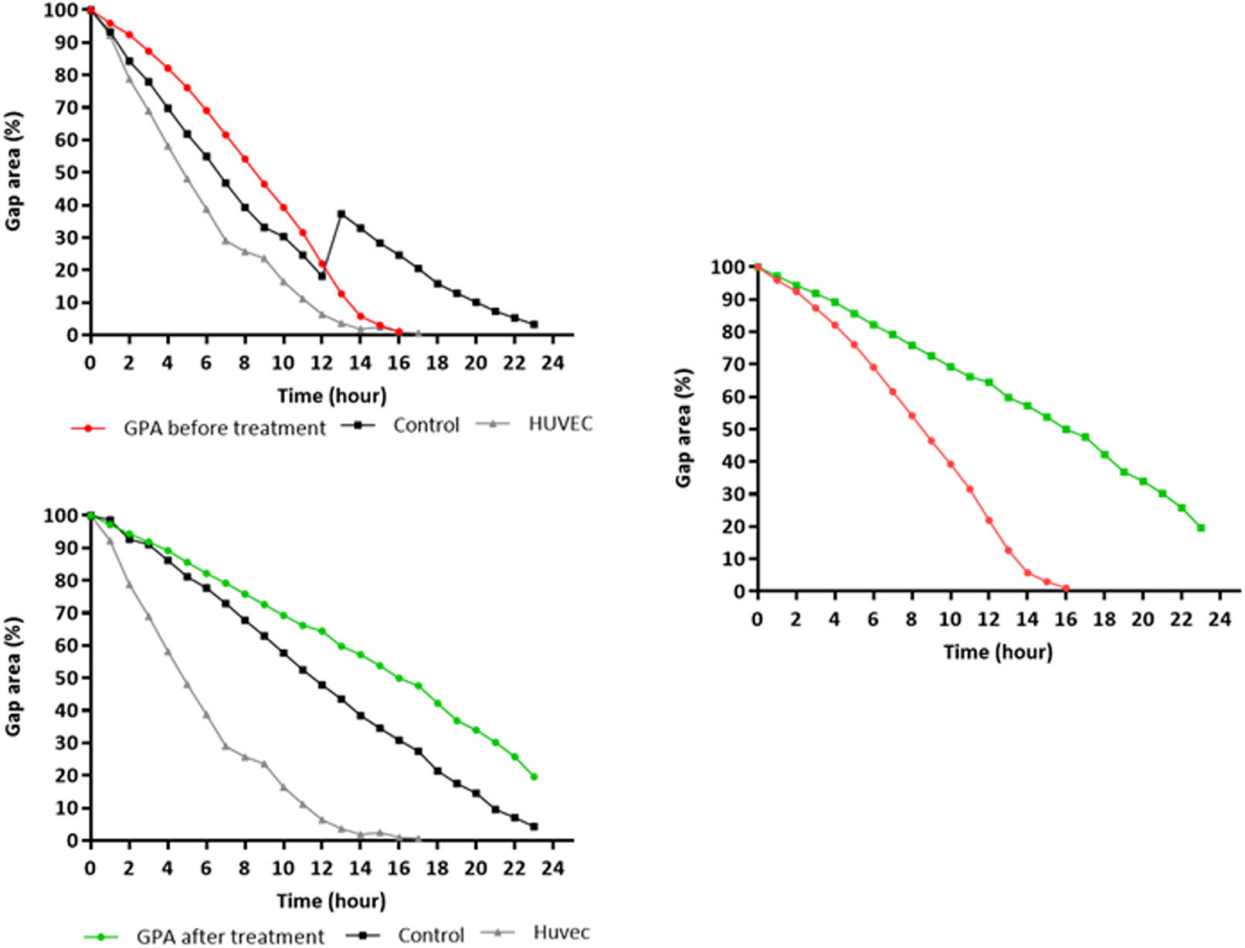
Longitudinal analysis: migration assay of ECFCs from a GPA patient before and after treatment, showing reduced proliferative capacity after remission.

Blood samples from three patients with GPA were collected to analyse the behaviour of ECFCs before and after treatment. All three patients exhibited glomerulonephritis and tested positive for PR3‐ANCA. Successful ECFC isolation was observed in only one patient after remission induction with intravenous cyclophosphamide. ECFCs isolated after cyclophosphamide treatment showed significantly lower displacement rates than those isolated before treatment (*p* = 0.0056) (Figure 5).

### 3.6 Influence of plasma on migration in vitro

To analyse the influence of plasma on the migration capacities of ECFCs, we performed scratch assays on ECFCs incubated overnight with plasma from healthy controls and patients with active disease. By comparing the lowest percentages of gap areas reached in 24 h between the groups (active GPA and GPA in remission, control and HUVECs) in both plasma conditions, the least displacement capacity was observed in the samples of GPA patients in remission when incubated with plasma from healthy controls (*p* = 0.0020).

**FIGURE 6:**
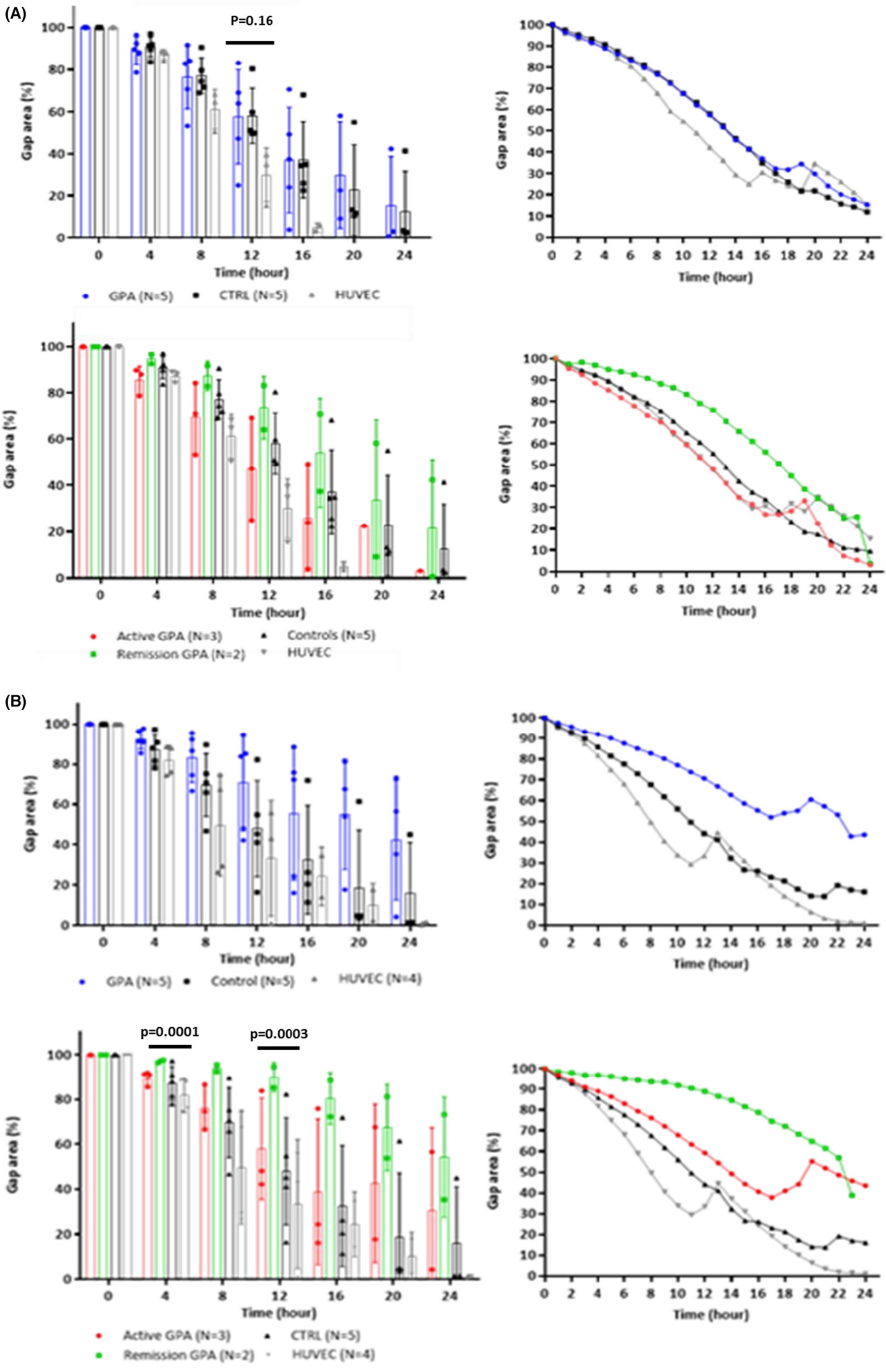
Measurement of cell migration in in vitro scratch assay after plasma stimulus: (A) After overnight incubation with 10% plasma from patients with active GPA: there were no significant differences in migration capacities, between ECFCs of controls and patients with GPA, even when considering active disease and cases in remission. There was a similar confluence behaviour considering individual samples and means of the groups. (B) After overnight incubation with 10% plasma from controls: cells from patients in remission exhibited significantly lower migration capacities than controls after the 4th hour.

The migration assay values of each group (active GPA, remission GPA, control and HUVECs) were also compared every 4 h until the gap area closed. When incubated with plasma from patients with active disease, decreased migration capacity, which was not statistically significant, was observed in the ECFCs of patients with GPA compared with those of the controls (12th h, *p* = 0.16; 16th h, *p* = 0.36). In addition, a higher migration capacity, which was not statistically significant, was observed in the ECFC subgroup of patients with active disease (remission, *p* = 0.31; control, *p* = 0.74). Considering the influence of the control plasma, the ECFCs of patients with GPA in remission showed significantly lower migration capacities after the 4th h (*p* = 0.0001) (Figure 6).

## 4 DISCUSSION

This study is the first to evaluate the angiogenic capacity of ECFCs from AAV patients. We aimed to evaluate endothelial cells of patients with PR3‐positive GPA using in vitro ECFC angiogenesis assays. Considering the recurrent nature of the disease, investigation of these mechanisms may lead to a better understanding of the perpetuation of endothelial injury and involvement of inadequate repair. This study showed a decrease in the ability to form capillary‐like structures and an alteration to the in vitro migration capacity of ECFCs from patients with GPA in remission compared with controls.

In patients with ANCA‐positive AAV, interactions between neutrophils and endothelial cells causes endothelial damage and imbalance.^3^ Angiogenesis, the formation of new blood vessels from pre‐existing ones, is a marker of vascular repair that depends on the migration and proliferation of endothelial cells. These mechanisms are highly influenced by plasma factors, adhesion molecules and hypoxia.^29^, ^30^, ^31^, ^32^ However, little is known regarding the repair mechanisms of the endothelial microcirculation in AAV. Previous studies have also reported that the number of circulating EPCs in AAV patients varies according to disease activity, allowing for the prediction of occurrence of relapses.^7^, ^33^, ^34^ Other studies have found a deterioration in the proliferative ability and growth of EPCs in culture. However, these studies performed assays on colony‐forming units‐endothelial cells (CFU‐ECs), which have no capacity for neovasculogenesis.^15^, ^19^, ^21^ Additionally, in a study by Patschan et al.^19^ greater PR3 expression was found to be associated with diminished proliferation.

In this study, we used ECFCs because they are progenitor cells with robust clonogenic potential that are suitable for ex vivo analysis of endothelial function. Thus, it is imperative to identify possible changes in the behaviour of these cells to enable potential future interventions. Successful isolation and cultivation of ECFCs were achieved, with yields ranging from 50% to 80%, which is consistent with previous studies depending on the protocols used.^14^, ^35^ In this study, cultures were successful in eight (62%) patients with GPA and eight (57%) controls.

Wilde et al.^21^ were the first to observe decreased differentiation and proliferation capacity in ECFC cultures from patients with AAV. This study also observed reduced ECFC growth capacity in patients with relapsing disease. However, ECFC differentiation and proliferation capacity were not impaired in patients with GPA, which contrasts with the findings of Wilde et al.^35^ in that patients with active disease had higher numbers of colonies, raising the average of the GPA group. While the mean age of the patients with GPA was higher than that of the controls, previous studies have stated that age has no influence on culture yields. In the present study, only patients with PR3‐positive‐ANCA were evaluated. Furthermore, patients with active disease were newly diagnosed and not previously treated, whereas those in remission were not immunosuppressed for >24 months. These differences in study populations may explain the discrepancies identified with those of the previous studies.

Although the difference was not statistically significant, we noticed a mild increase in the number of in vitro tubes formed in the Matrigel assay of ECFCs from patients with GPA compared with controls. Interestingly, the number of capillary structures formed by ECFCs from patients with inactive GPA decreased between 15 and 24 h, showing an early loss of angiogenic capacity and derangements involving vascular structures. Although impaired angiogenic function has already been reported by Holmén et al.^20^ in CFU‐ECs from patients with GPA, this is the first study to observe it in ECFCs.

In the scratch assay, the ECFC migration capacities in patients with GPA were similar to those in control patients and HUVECs. The influence of cytokines and growth factors on ECFC function was replicated by performing migration experiments using plasma from healthy controls and patients with active GPA. Incubation with both types of plasma reduced the migration capacity of ECFCs from patients with GPA in remission compared with ECFCs from controls. Interestingly, significant differences were observed only in those incubated with healthy control plasma. Although the exact influence of plasma on the cells of patients in remission is unknown, these findings support previous findings of microvascular endothelial dysfunction that may contribute to poor vascular repair.^12^ Furthermore, persistent vascular damage may favour continuous immune activation and recurrence.^7^, ^37^

Longitudinal assessment of one patient before and after 6 months of treatment with corticosteroids and cyclophosphamide revealed a lower ECFC migration rate and reduced migration capacity. Thus, interference by immunosuppression may have influenced growth despite the successful isolation of several colonies in this case. This is consistent with previously described results from the cells of patients in remission.

The main limitations of this study included the difficulty in recruiting a large number of patients due to the monocentric design of the study, the rarity and severity of the disease that often requires prompt immunosuppression, and the difficulty in successfully growing ECFCs. While in vitro assays are known to be an accessible and reliable model for investigating the role of ECFCs in angiogenesis, selecting the ideal methodology remains challenging owing to possible limitations in terms of interpretation of these findings.

This study took an initial step towards an improved understanding of the course of ECFCs undergoing angiogenesis. We revealed the possibility of intrinsic endothelial cell changes in patients with GPA, compromising their angiogenic capacity. By evaluating capillary formation and performing scratch assays, ECFCs in patients with GPA in remission exhibited decreased angiogenesis compared with controls. Thus, we hypothesized that impairment of vascular repair function in patients with anti‐PR3‐positive GPA may be associated with its recurrent nature. Future studies are needed to identify factors that may improve vascular homeostasis in patients with GPA.

## 5 CONCLUSION

In summary, ECFCs from patients with PR3‐positive GPA in remission demonstrated impairment of angiogenic function, as observed from decreased tube capillary formation and migration capacity compared with those of the healthy controls, suggesting altered endothelial function. These findings highlight the need for further studies concerning endothelial function in patients with GPA, which may lead to the development of new therapeutic approaches.

## AUTHOR CONTRIBUTIONS

**Ana Paula Toledo Del Rio:** Conceptualization (equal); data curation (equal); formal analysis (equal); investigation (equal); methodology (equal); project administration (equal); validation (equal); visualization (equal); writing – original draft (equal); writing – review and editing (equal). **Jéssica O Frade‐Guanaes:** Conceptualization (equal); data curation (equal); formal analysis (equal); methodology (equal); software (equal); writing – original draft (supporting). **Stephanie Ospina‐Prieto:** Conceptualization (equal); formal analysis (equal); methodology (equal); software (supporting); validation (supporting). **Bruno K L Duarte:** Conceptualization (supporting); data curation (supporting); formal analysis (supporting); software (supporting); writing – original draft (supporting); writing – review and editing (supporting). **Manoel Barros Bertolo:** Supervision (equal); writing – original draft (supporting); writing – review and editing (supporting). **Margareth C Ozelo:** Conceptualization (equal); funding acquisition (lead); investigation (supporting); methodology (supporting); project administration (supporting); resources (supporting); software (supporting); supervision (supporting); validation (supporting); visualization (supporting); writing – original draft (supporting); writing – review and editing (supporting). **Zoraida Sachetto:** Conceptualization (lead); data curation (lead); formal analysis (lead); investigation (lead); methodology (equal); project administration (lead); resources (equal); supervision (lead); validation (lead); visualization (lead); writing – original draft (equal); writing – review and editing (equal).

## CONFLICT OF INTEREST

The authors declare no competing interests.

## Data Availability

The datasets analysed in this study are available from the corresponding author upon reasonable request.

## References

[1] Jennette JC , Falk RJ , Bacon PA , et al. 2012 revised international Chapel Hill consensus conference nomenclature of vasculitides. Arthritis Rheum. 2013;65(1):1‐11. 23045170 10.1002/art.37715

[2] Flint J , Morgan MD , Savage CO . Pathogenesis of ANCA‐associated vasculitis. Rheum Dis Clin North Am. 2010;36(3):463‐477. 20688244 10.1016/j.rdc.2010.05.006 PMC3486518

[3] Al‐Hussain T , Hussein MH , Conca W , Al Mana H , Akhtar M . Pathophysiology of ANCA‐associated vasculitis. Adv Anat Pathol. 2017;24(4):226‐234. 28537941 10.1097/PAP.0000000000000154

[4] Morgan MD , Turnbull J , Selamet U , et al. Increased incidence of cardiovascular events in patients with antineutrophil cytoplasmic antibody‐associated vasculitides: a matched‐pair cohort study. Arthritis Rheum. 2009;60(11):3493‐3500. 19877070 10.1002/art.24957

[5] Almaani S , Fusnner LA , Brodsky S , Meara AS , Jayne D . ANCA associated vasculitis: an update. J Clin Med. 2021;10:1446. 33916214 10.3390/jcm10071446 PMC8037363

[6] Kong AM , Kim G , Michalska M , Best JH . Costs of disease relapse among individuals with granulomatosis, with poliangiitis or microscopic poliangiitis in the United States. Rheumatol Ter. 2018;5(1):159‐170. 10.1007/s40744-018-0099-1 PMC5935630 29470835

[7] Závada J , Kideryová L , Pytlík R , Hrusková Z , Tesar V . Reduced number of endothelial progenitor is predictive of early relapse in anti‐neutrophyl cytoplasmic antibody‐associated vasculitis. Rheumatology (Oxford). 2009;48(10):1197‐1201. 19509188 10.1093/rheumatology/kep130

[8] Asahara T , Murohara T , Sullivan A , et al. Isolation of putative progenitor endothelial cell for angiogenesis. Science. 1997;275:964‐967. 9020076 10.1126/science.275.5302.964

[9] Yoder MC , Mead LE , Prater D , et al. Redefining endothelial progenitor cells via clonal analysis and hematopoietic stem/progenitor cell principals. Blood. 2007;109(5):1801‐1809. 17053059 10.1182/blood-2006-08-043471 PMC1801067

[10] Medina RJ , Barber CL , Sabatier F , et al. Endothelial progenitors: a consensus Statement on nomenclature. Stem Cells Transl Med. 2017;6:1316‐1320. 28296182 10.1002/sctm.16-0360 PMC5442722

[11] Chambers SEJ , Pathak V , Pedrini E , et al. Current concepts on endothelial stem cells definition, location, and markers. Stem Cells Transl Med. 2021;10:S54‐S61. 34724714 10.1002/sctm.21-0022 PMC8560200

[12] Ferrante A , Guggino G , di Liberto D , et al. Endothelial progenitor cells: are they displaying a function in autoimmune disorders? Mech Ageing Dev. 2016;159:44‐48. 27153975 10.1016/j.mad.2016.05.001

[13] Alphonse RS , Vadivel A , Zhong S , et al. The isolation and culture of endothelial colony‐forming cells from human and rat lungs. Nat Protoc. 2015;10:1697‐1708. 26448359 10.1038/nprot.2015.107

[14] Smadja DM , Melero‐Martin JM , Eikenboom J , Bowman M , Sabatier F , Randi AM . Standardization of methods to quantify and culture endothelial colony‐forming cells derived from peripheral blood: position paper from the international society on thrombosis and Haemostasis SSC. J Thromb Haemost. 2019;17(7):1190‐1194. 31119878 10.1111/jth.14462 PMC7028216

[15] Critser PJ , Yoder MC . Endothelial Colony forming cell role in neoangiogenesis and tissue repair. Curr Opin Organ Transplant. 2010;15(1):68‐72. 19898235 10.1097/MOT.0b013e32833454b5 PMC2880951

[16] Liao G , Zheng K , Shorr R , Allan DS . Human endothelial colony‐forming cells in regenerative therapy: a systematic review of controlled preclinical animal studies. Stem Cells Transl Med. 2020;9:1344‐1352. 32681814 10.1002/sctm.20-0141 PMC7581447

[17] Woywodt A , Streiber F , de Groot K , Regelsberger H , Haller H , Haubitz M . Circulating endothelial cells as markers for ANCA‐associated small‐vessel vasculitis. Lancet. 2003;361(9353):206‐210. 12547543 10.1016/S0140-6736(03)12269-6

[18] Závada J , Kideryová L , Pytlík R , Vanková Z , Tesar V . Circulating endothelial progenitor cells in patients with ANCA‐associated vasculitis. Kidney Blood Press Res. 2008;31(4):247‐254. 18600027 10.1159/000142690

[19] Patschan S , Patschan D , Henze E , Blaschke S , Wessels JT , Müller GA . Impairment and differential expression of PR3 and MPO on peripheral myelomonocytic cells with endothelial properties in granulomatosis with polyangiitis. Int J Nephrol. 2012;2012:715049. 22792461 10.1155/2012/715049 PMC3390043

[20] Holmén C , Elsheikh E , Stenvinkel P , et al. Circulating inflammatory endothelial cells contribute to endothelial progenitor cell dysfunction in patients with vasculitis and kidney involvement. J Am Soc Nephrol. 2005;16(10):3110‐3120. 16107582 10.1681/ASN.2005040347

[21] Wilde B , Mertens A , Arends SJ , et al. Endothelial progenitor cell are differentially impaired in ANCA associated vasculitis compared with health controls. Arthritis Res Ther. 2016;18:147. 27338585 10.1186/s13075-016-1044-8 PMC4918016

[22] Luqmani RA et al. Birmingham Vasculitis Activity Score (BVAS) in systemic necrotizing vasculitis. QJM. 1994;87(11):671‐678. 7820541

[23] Stone JH , Hoffman GS , Merkel PA , et al. A disease‐specific activity index for Wegener's granulomatosis: modification of the Birmingham Vasculitis activity score. International network for the Study of the Systemic Vasculitides (INSSYS). Arthritis Rheum. 2001;44(4):912‐920. 11318006 10.1002/1529-0131(200104)44:4<912::AID-ANR148>3.0.CO;2-5

[24] Hellmich B , Flossmann O , Gross WL , et al. EULAR recommendations for conducting clinical studies and/or clinical trials in systemic vasculitis: focus on anti‐neutrophil cytoplasm antibody‐associated vasculitis. Ann Rheum Dis. 2007;66(5):605‐617. 17170053 10.1136/ard.2006.062711 PMC2703775

[25] Lin Y , Weisdorf DJ , Solovey A , Hebbel RP . Origins of circulating endothelial cells and endothelial outgrowth from blood. J Clin Invest. 2000;105(1):71‐77. 10619863 10.1172/JCI8071 PMC382587

[26] Sakamoto TM , Lanaro C , Ozelo MC , et al. Increased adhesive and inflammatory properties in blood outgrowth endothelial cells from sickle cell anemia patients. Microvasc Res. 2013;90:173‐179. 24144783 10.1016/j.mvr.2013.10.002

[27] Ferratge S , Ha G , Carpentier G , et al. Initial clonogenic potential of human endothelial progenitor cells is predictive of their further properties and establishes a functional hierarchy related to immaturity. Stem Cell Res. 2017;21:148‐159. 28499264 10.1016/j.scr.2017.04.009

[28] Liang CC , Park AY , Guan JL . In vitro scratch assay: a convenient and inexpensive method for analysis of cell migration in vitro. Nat Protoc. 2007;2(2):329‐333. 17406593 10.1038/nprot.2007.30

[29] Shweiki D , Itin A , Soffer D , Keshet E . Vascular endothelial growth factor induced by hypoxia may mediate hypoxia‐initiated angiogenesis. Nature. 1992;359(6398):843‐845. 1279431 10.1038/359843a0

[30] Patel J , Seppanen EJ , Rodero MP , et al. Functional definition of progenitors versus mature endothelial cells reveals key SoxF‐dependent differentiation process. Circulation. 2017;135(8):786‐805. 27899395 10.1161/CIRCULATIONAHA.116.024754

[31] Nowak‐Sliwinska P , Alitalo K , Allen E , et al. Consensus guidelines for the use and interpretation of angiogenesis assay. Angiogenesis. 2018;21(3):425‐532. 29766399 10.1007/s10456-018-9613-x PMC6237663

[32] Unterleuthner D , Neuhold P , Schwarz K , et al. Cancer‐associated fibroblast‐derived WNT2 increases tumor angiogenesis in colon câncer. Angiogenesis. 2020;23(2):159‐177. 31667643 10.1007/s10456-019-09688-8 PMC7160098

[33] De Groot K et al. Vascular endothelial damage and repair in antineutrophil cytoplasmic antibody‐associated vasculitis. Arthritis Rheum. 2007;56(11):3847‐3853. 17968939 10.1002/art.23070

[34] Santana ANC . Circulating endothelial progenitor cells in ANCA‐associated vasculitis: the light at the end of the tunnel? Rheumatology (Oxford). 2009;48(10):1183‐1184. 19620210 10.1093/rheumatology/kep216

[35] Rignault‐Clerc S , Bielmann C , Delodder F , et al. Functional late outgrowth endothelial progenitors isolated from peripheral blood of burned patients. Burns. 2012;39(4):694‐704. 23102579 10.1016/j.burns.2012.09.027

[36] Zhou Z , Han H , Cruz M , López J , Dong JF , Guchhait P . Haemoglobin blocks von Willebrand fator proteolysis by ADAMTS‐13: a mechanism associated with sickle cell disease. Thromb Haemost. 2009;101(6):1070‐1077. 19492149

[37] Martin‐Ramirez J , Hofman M , van den Biggelaar M , Hebbel RP , Voorberg J . Establishment of outgrowth endothelial cells from peripheral blood. Nat Protoc. 2012;7(9):1709‐1715. 22918388 10.1038/nprot.2012.093

